# Supplementary material for: The interactome of intact mitochondria by cross-linking mass spectrometry provides evidence for coexisting respiratory supercomplexes
Source: Mol Cell Proteomics. 2017 Dec 8;17(2):216–32. doi: 10.1074/mcp.RA117.000470 (PMC5795388; doi:10.1074/mcp.RA117.000470)
Supplement: Supplemental Data [file supp_RA117.000470_133922_0_supp_23978_fzffwf.zip › spectra_annotation/mito_DR_spectra_annotation/76-2-2-1-2-1.pdf]

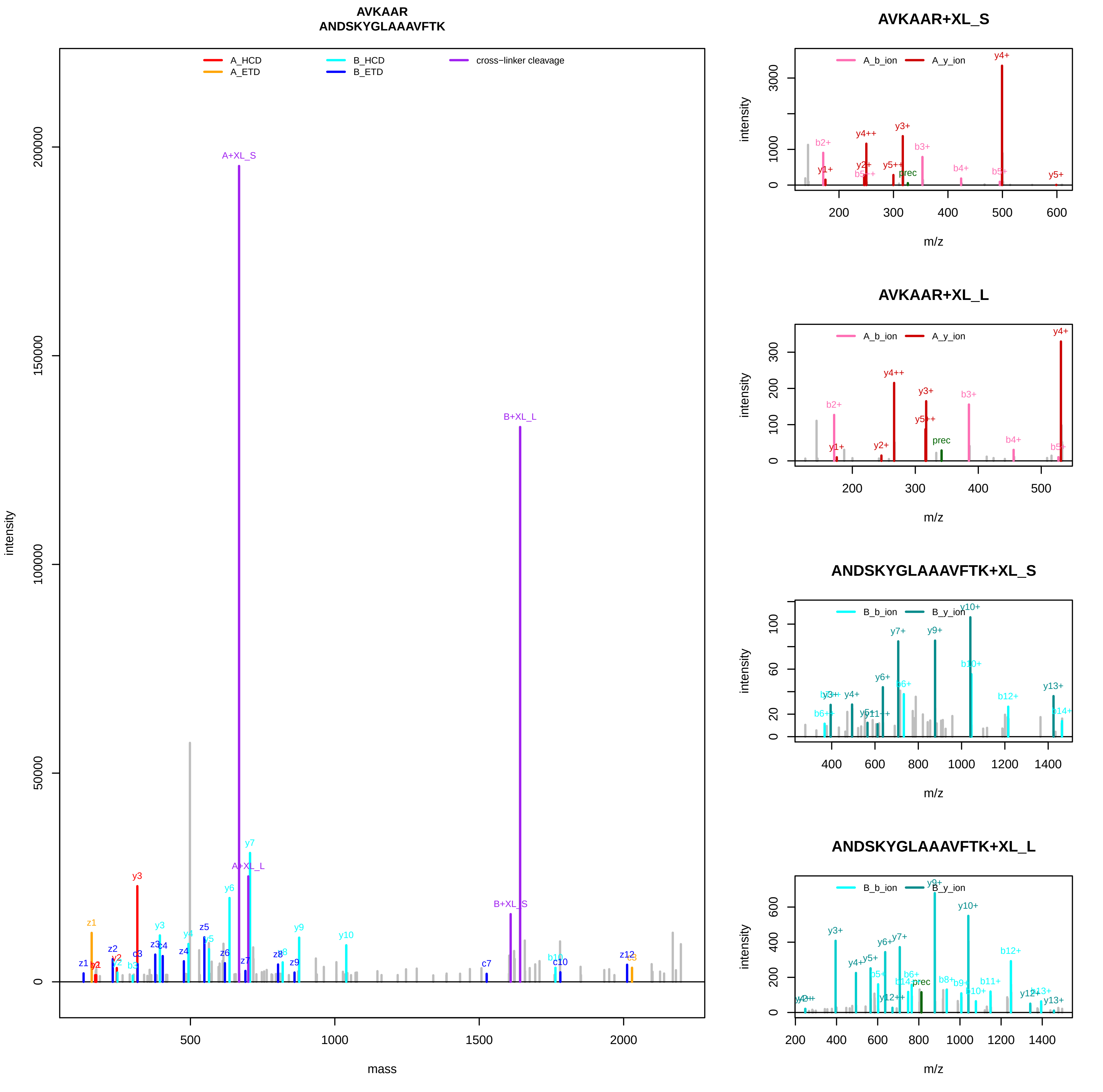

AVKAAR
ANDSKYGLAAAVFTK
A_HCD
A_ETD
B_HCD
B_ETD
cross−linker cleavage
intensity
mass
0
50000
100000
150000
200000
500
1000
1500
2000
A+XL_S
B+XL_L
A+XL_L
B+XL_S
y3
y6
y7
y9
y10
z1
z2
z3
z4
z5
z6
z7
z8
z9
z12
c3
c4
c7
c10
AVKAAR+XL_S
A_b_ion
A_y_ion
m/z
y4+
y4++
y3+
b2+
b3+
b4+
y1+
y2+
y5++
prec
y5+
AVKAAR+XL_L
ANDSKYGLAAAVFTK+XL_S
B_b_ion
B_y_ion
y10+
y7+
y9+
y6+
y13+
b12+
y4+
ANDSKYGLAAAVFTK+XL_L
y9+
y10+
y3+
y7+
y6+
y5+
y4+
b12+
b11+
y12+
y13+
